# Literary Notes

**Published:** 1900-09

**Authors:** 


### LITERARY NOTES.

THE Maltine Company, of Brooklyn, recently published a little book entitled Recipes for Infant Food. It contains a preface giving hints on the subject of infant feeding and each sheet, perforated to be easily detached, presents condensed and simple directions for preparing modified milk food with a blank space left for such special instructions as the physician may choose to give in each particular case. On the reverse more extended general directions are printed for the use of the same food. The book is bound in morocco and is of a size to be carried conveniently in the vest pocket. It will be furnished by the Maltine Company on application.

---

LISTERINE is the title of a summer pamphlet recently issued by the Lambert Pharmacal Company, of St. Louis. It deals with the summer diseases of infancy and childhood and contains a number of articles written by prominent members of the medical profession that have been contributed to different medical journals. These have been grouped, the whole forming a useful monograph that will be sent to any physician upon application to the publishers.

